# Age- and sex-specific associations of frailty with mortality and healthcare utilization in community-dwelling adults from Ontario, Canada

**DOI:** 10.1186/s12877-024-04842-4

**Published:** 2024-03-04

**Authors:** Chris P. Verschoor, Olga Theou, Jinhui Ma, Phyllis Montgomery, Sharolyn Mossey, Parveen Nangia, Refik Saskin, David W. Savage

**Affiliations:** 1 Health Sciences North Research Institute, Sudbury, ON Canada; 2 NOSM University, Sudbury/Thunder Bay, ON Canada; 3 Dept. of Health Research Methods, Evidence and Impact, McMaster University, Hamilton, ON Canada; 4 School of Physiotherapy, Dalhousie University, Halifax, NS Canada; 5 School of Nursing, Laurentian University, Sudbury, ON Canada; 6 School of Social Sciences, Laurentian University, Sudbury, ON Canada; 7 ICES, Toronto, ON Canada; 8 56 Walford Road, Rm. 119, P3E 2H2 Sudbury, ON Canada

**Keywords:** Aging, Frailty index, Mortality, Healthcare utilization, Hospital admission

## Abstract

**Background:**

Understanding how health trajectories are related to the likelihood of adverse outcomes and healthcare utilization is key to planning effective strategies for improving health span and the delivery of care to older adults. Frailty measures are useful tools for risk stratification in community-based and primary care settings, although their effectiveness in adults younger than 60 is not well described.

**Methods:**

We performed a 10-year retrospective analysis of secondary data from the Ontario Health Study, which included 161,149 adults aged ≥ 18. Outcomes including all-cause mortality and hospital admissions were obtained through linkage to ICES administrative databases with a median follow-up of 7.1-years. Frailty was characterized using a 30-item frailty index.

**Results:**

Frailty increased linearly with age and was higher for women at all ages. A 0.1-increase in frailty was significantly associated with mortality (HR = 1.47), the total number of outpatient (IRR = 1.35) and inpatient (IRR = 1.60) admissions over time, and length of stay (IRR = 1.12). However, with exception to length of stay, these estimates differed depending on age and sex. The hazard of death associated with frailty was greater at younger ages, particularly in women. Associations with admissions also decreased with age, similarly between sexes for outpatient visits and more so in men for inpatient.

**Conclusions:**

These findings suggest that frailty is an important health construct for both younger and older adults. Hence targeted interventions to reduce the impact of frailty before the age of 60 would likely have important economic and social implications in both the short- and long-term.

**Supplementary Information:**

The online version contains supplementary material available at 10.1186/s12877-024-04842-4.

## Introduction

Between 2021 and 2068 it is estimated that the proportion of older adults in Canada will increase from approximately 18.5% to as much as 30% [1]. This rapid growth will require significant investment in health and social services in order to effectively prepare for and respond to their unique needs. Given the scale of this challenge and the impact it will have on societies from an economic and even cultural perspective, there is greater emphasis towards lifestyle choices and health interventions that promote what is colloquially known as “healthy aging”, maximizing the years one lives without being burdened by chronic illness or suboptimal well-being. Although primary care approaches tend to focus on the treatment of individual diseases, there is a groundswell of evidence suggesting that the most effective interventions would be those that improve overall health as we age. In fact, a recent modelling study found that reducing the number of years lived with multiple chronic conditions would yield greater economic value than eradicating any single condition including cancer, dementia or cardiovascular diseases alone, with an overall impact in the tens of trillions of dollars per year in the United States [2]. What remains unknown is the age at which healthy aging interventions would be most effective in improving overall healthspan and reducing healthcare burden.

Frailty is an age-related syndrome that in many ways is the antithesis to healthy aging. It is considered a state of vulnerability and compromised resilience to stressors and as such, is broadly related to adverse health outcomes in a variety of settings [3]. For example, it is widely used in critical care to identify patients at greater risk of complications during treatment and post-discharge outcomes [4], in community settings to identify individuals who likely require long-term care [5] and as a risk stratification tool for older cancer patients [6]. One of the most common approaches to estimating frailty is by the deficit accumulation model, wherein the presence of a variety of health aspects spanning illness, disability, physical and cognitive function, mental health and/or well-being is used to derive a continuous score known as the frailty index. Although it is often considered a geriatric syndrome, frailty measured using a frailty index is detectable in all adults and is a significant predictor of mortality as young as 20–30 years old [7]. However, while the risk of death associated with frailty appears to be stable across studies and over time [8], some studies suggest that it may also decline with age and be greater in men than women [7, 9]. There is also evidence showing frailty to be associated with healthcare utilization over time, including hospital admissions, length of stay and long-term care services [10, 11]. For example, physical frailty was shown to be associated with 1.3-times longer length of inpatient stay and 4.4-times higher risk of long-term care service use in Korean adults over 65 [10], and a 1.30- and 1.44-times higher risk of outpatient and inpatient admission, respectively, in Chinese adults 60 and older [11]. However, it is unclear how this risk profile changes with age given that most studies are performed in relatively homogenous, older and often disease-specific populations.

In the following study, we sought to estimate the association of frailty with the risk of mortality, incidence of hospital admissions, and inpatient length of stay in a cohort of community-dwelling adults aged 18 and older residing in Ontario, Canada. Analyses were performed on the entire cohort (*n* = 161,149) as well as within age- and sex-strata for a median follow-up period of 7.1 years.

## Methods

### Study design

We conducted a longitudinal analysis of secondary data from the Ontario Health Study (OHS) [12]. The OHS is a cohort study of 225,620 adults aged 18 and older recruited between 2009 and 2017 from the province of Ontario, Canada. Study eligibility criteria included proficiency in English or French, provision of informed consent, and access to the internet for questionnaire completion. Online survey questions elicited data regarding sociodemographics, family history, health status, behavioural factors, lifestyle factors and self-reported anthropometry. All participants provided information at baseline (2009–2017), while some also provided information at a follow-up collection (2016–2019); follow-up data collected by the OHS were not considered for this study. Study outcomes were obtained through linkage to administrative data holdings of ICES (formerly the Institute of Clinical and Evaluative Sciences) [13] using its Data and Analytic Services platform. Specifically, linkage was performed to the Ontario Registered Persons Database (RPDB) and the Discharge Abstract Database (DAD), which includes administrative, clinical, and demographic information on all hospital admissions and discharges in the province. The current study focused on 178,874 unique participants aged 18 and older that provided baseline data to the OHS between 2009 and 2013 and could be linked to ICES databases; no additional exclusion criteria were applied. Follow-up in ICES databases was performed until December 31st, 2019. The study and its protocol was approved by the Health Sciences North Research Ethics Board (#21 − 007).

### Sociodemographic and lifestyle characteristics

Participants were characterized according to age, sex, ethnicity, marital status, education, total household income, geography, smoking behaviours and alcohol consumption. Age was categorized according to decile (i.e. 18–29, 30–39, 40–49, 50–59, 60–69, 70–79, and 80+) to reduce the opportunity for participant identification as per ICES privacy policies. Ethnicity was categorized according to self-identification as white or not-white. Geography was classified as either urban or rural, determined by the second digit of the forward sortation area of the participant’s postal code. Region was further classified as north or south, according to the Local Health Integration Network (LHIN) in which the participants resided (i.e. north encompassed Northeastern and Northwestern Ontario; whereas, south encompassed all other LHINs). Alcohol consumption was categorized relative to number of drinks per month or week. Smoking was categorized as history (i.e. never, former or current) and pack-years exposure (i.e. less than 10, or 10 or more).

### Frailty index

A frailty index was developed according to published guidelines [14] using 30 deficit items encompassing self-rated health and lifestyle related risk factors, physical activity, disability and chronic conditions and related risk factors (Supplemental Table 1). Specifically, this included: self-rated general health and vision, body-mass index, average sleep time and trouble sleeping, living alone, frequency walking and performing moderate or vigorous activities, the inability to work due to illness or stand without assistance, ever receiving radiotherapy or chemotherapy, a family history of dementia and medication use. Self-reported chronic conditions included: arthritis, asthma, cancer, heart disease, chronic obstructive pulmonary disease, Crohn’s disease, depression, diabetes, high blood pressure, irritable bowel syndrome, heart attack, multiple sclerosis, osteoporosis, Parkinson’s disease, stroke and ulcerative colitis. The frailty index (FI) was assigned as missing for any participant that was missing 6 or more items (≥ 20%), and considered as both a continuous and categorical measure (i.e. low [FI ≤ 0.1], mild [0.1 < FI ≤ 0.2], moderate [0.2 < FI ≤ 0.3] and high [FI > 0.3]). Generally, the frailty index ranges between 0 and 0.65 (commonly observed submaximal), with a reported reliability (intraclass correlation) of 0.88 [15].

### Outcomes

Study outcomes included all-cause mortality, hospital admissions, and length of stay for inpatient visits. Mortality was obtained from the RPDB as the time to death in days relative to the date that the baseline questionnaire was completed; for example, a participant who completed their questionnaire on January 1st, 2010 and passed away December 31st, 2016 would have a time to death of 2556 days. Hospital admissions were obtained from the DAD and recorded as the time to occurrence relative to the date that the baseline questionnaire was completed. Participants were categorized as either outpatient (admitted to hospital for treatment and discharged the same day) or inpatient (admitted to hospital for urgent or elective treatment and stayed overnight) visits and analyzed separately. For mortality, participants were censored at the date of the most recent record available in any of the ICES databases employed. The length of stay in days for any single inpatient admission record was also obtained from the DAD.

### Statistical analysis

Participant characteristics were summarized as the mean and standard deviation and/or median and quantiles for continuous data and count and frequency for categorical data. Mortality and hospital admissions were summarized as the mean cumulative incidence and the total number observed, and length of stay as the mean and standard deviation of the within patient length of stay geometric mean; the geometric mean was used given that length of stay data is commonly right-skewed. The mean cumulative incidence of admissions was calculated using the R package ‘reda’ (https://github.com/wenjie2wang/reda). This package facilitates the exploration and modeling of recurrent event data by employing the mean cumulative function. Face validity of the frailty index was assessed by comparing distribution characteristics (e.g. mean, variance, range) to that obtained for similar cohorts and by estimating associations to participant sociodemographic and lifestyle characteristics that have been previously shown to correlate with frailty. This was done by way of a generalized linear model and expressed as the crude or adjusted coefficient and 95% confidence interval (CI); all participant characteristics were included in the adjusted model given our previous work showing that they are associated with frailty [16] and other studies indicating their importance to health outcomes. Associations of the frailty index with mortality were estimated using Cox proportional hazards models and presented as crude and adjusted hazards ratios (HR) and 95% CI. Associations with the total number of hospital admissions observed were estimated using Poisson regression and included a logged offset to account for differences in follow-up period; these were presented as incidence rate ratios (IRR) and 95% CI. For the length of stay of inpatient admissions, associations were estimated using generalized estimating equation-based negative binomial models with an exchangeable correlation structure and clustered according to participant. Performed using the R package “geeM” [17], this was only conducted for those with inpatient records, and was presented as the IRR and 95% CI. For all outcome models, crude and adjusted estimates were calculated in the entire sample and within age- and sex-specific strata relative to a 0.1-increase in the frailty index. Covariates in adjusted models were determined empirically as those that improve the model fit (i.e. adjusted R-squared) in the entire sample when included alongside the frailty index.

All analyses were performed in R v4.2.2.

## Results

### Participant characteristics and the frailty index

Of the 178,874 participants that were considered for this study, the frailty index was missing for 17,725 individuals. The resulting 161,149 participants comprised our analytic sample (Table 1). Participants were predominantly women (60%), self-reported as white (82%), resided in urban communities (89%), and never smoked (55%). The largest age group 50–59 years (24%) and those participants aged 60 years or older comprised 22% of the overall sample. The median follow-up was 7.10 years for all participants with exception of the 80 years and older group, for which it was 7.05 years.

**Table 1:** Characteristics off the 161,149 participants of the Ontario Health Study that were included for analysis

Age	18–29	26,070 (16.2%)
	30–39	27,473 (17.0%)
	40–49	33,177 (20.6%)
	50–59	38,499 (23.9%)
	60–69	27,150 (16.8%)
	70–79	7462 (4.6%)
	80+	1318 (0.8%)
Sex	Female	96,857 (60.1%)
	Male	64,292 (39.9%)
Ethnicity	Not white	25,890 (16.1%)
	White	131,452 (81.6%)
	Missing	3807 (2.4%)
Marital	Never married	30,897 (19.2%)
	Previously married	22,329 (13.9%)
	Married	105,725 (65.6%)
	Missing	2198 (1.4%)
Education	Less than diploma	45,454 (28.2%)
	Diploma	43,943 (27.3%)
	Bachelor	44,594 (27.7%)
	Graduate	25,148 (15.6%)
	Missing	2010 (1.2%)
Total household income	Less than 50 K	39,902 (24.8%)
	50-100 K	51,837 (32.2%)
	100-150 K	28,298 (17.6%)
	150 K+	21,043 (13.1%)
	Missing	20,069 (12.5%)
Geography	Urban	143,359 (89.0%)
	Rural	17,457 (10.8%)
	Missing	333 (0.2%)
Region	South	151,894 (94.3%)
	North	9255 (5.7%)
Alcohol consumption	< 1/Month	46,902 (29.1%)
	1–3/Month	34,202 (21.2%)
	1–3/Week	47,250 (29.3%)
	4–7/Week	31,089 (19.3%)
	Missing	1706 (1.1%)
Smoking status [Pack years]	Never	89,160 (55.3%)
	Former [< 10]	29,096 (18.1%)
	Current [< 10]	10,593 (6.6%)
	Former [10+]	18,957 (11.8%)
	Current [10+]	11,542 (7.2%)
	Missing	1801 (1.1%)

**Fig. 1:**
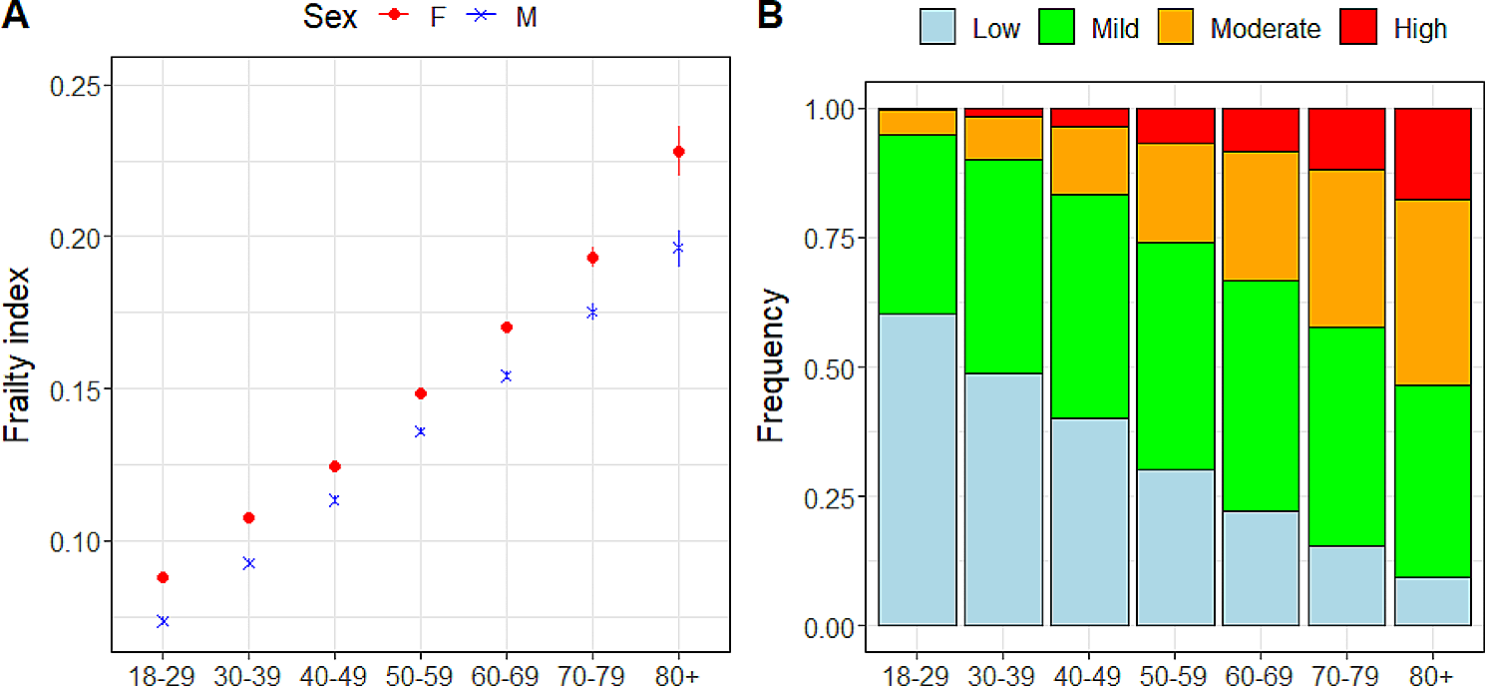
A summary of the frailty index in the sample population, stratified by age and sex. **A**) The mean and standard error of frailty in women and men across age group, and **B**) the proportion of frailty categories across ages

The mean frailty index for the entire cohort was 0.140 ± 0.085, with a 1st and 99th percentile of 0 and 0.397, respectively. As expected, frailty increased with age in a linear fashion and was significantly greater in women, regardless of age (Fig. 1A). When considered categorically, 38% were low (FI ≤ 0.1), 42% were mild (0.1 < FI ≤ 0.2), 15% were moderate (0.2 < FI ≤ 0.3) and 5% were high (FI > 0.3); both the high and moderate groups increased with age, while the low group decreased (Fig. 1B). Relative to sociodemographic- and lifestyle-related characteristics, trends for the frailty index were also as expected (Supplemental Table 2). In the fully adjusted model (adjusted r^2^ = 0.235 for 134,776 complete cases), frailty was significantly lower in males, individuals that are or have been married, those who have received a diploma or higher education, income greater than $50,000, rural residence, those who consume alcohol more than once per month and never smoked; dose dependant relationships were apparent where applicable. In contrast, frailty was significantly higher in older adults, residents in Northern Ontario and those self-identified as white.

### Associations between frailty and all-cause mortality

**Fig. 2:**
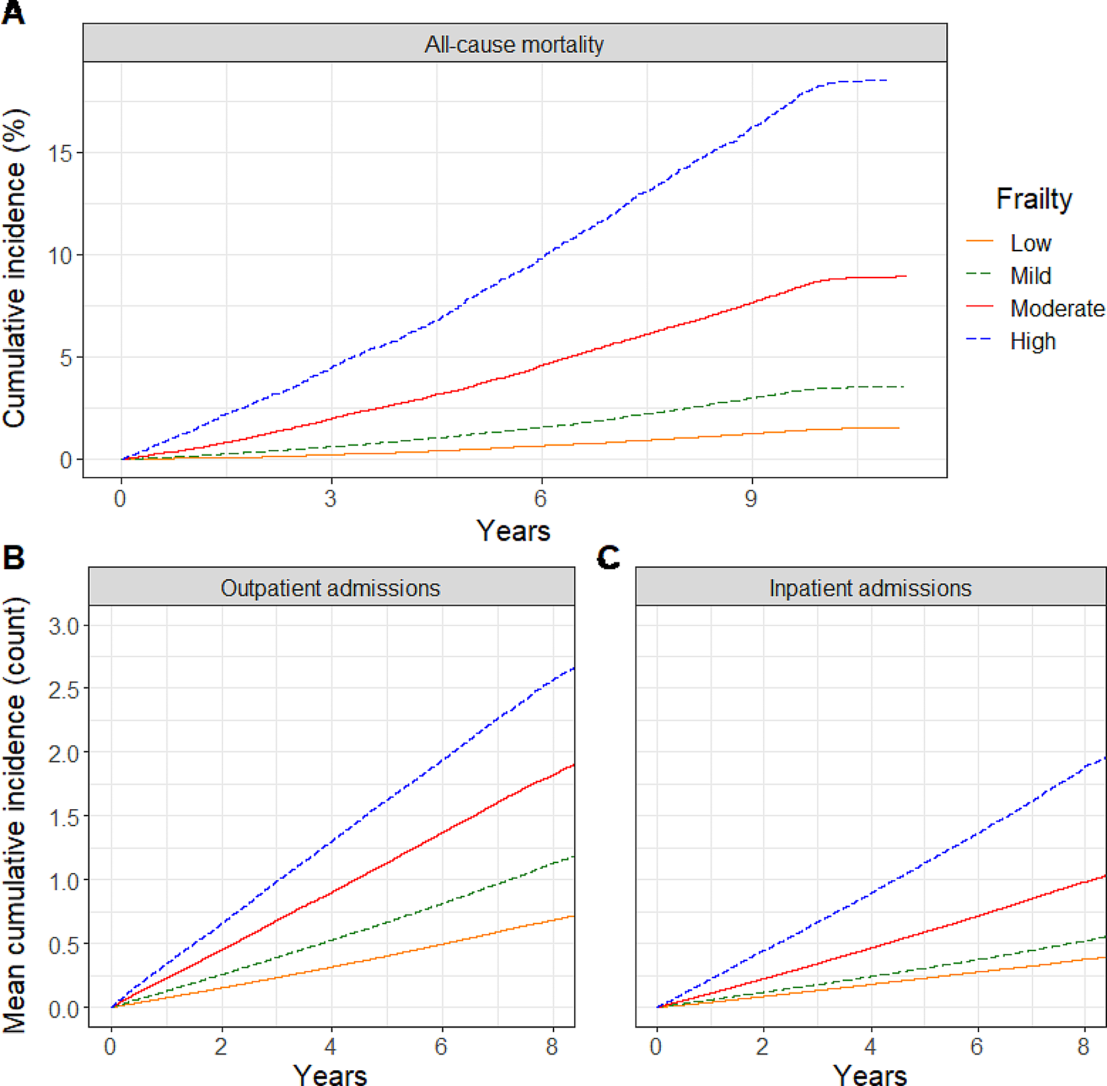
Cumulative incidence of health outcomes over the follow-up period, stratified by age group. **A**) All-cause mortality, **B**) outpatient admissions, and **C**) inpatient admissions

**Fig. 3:**
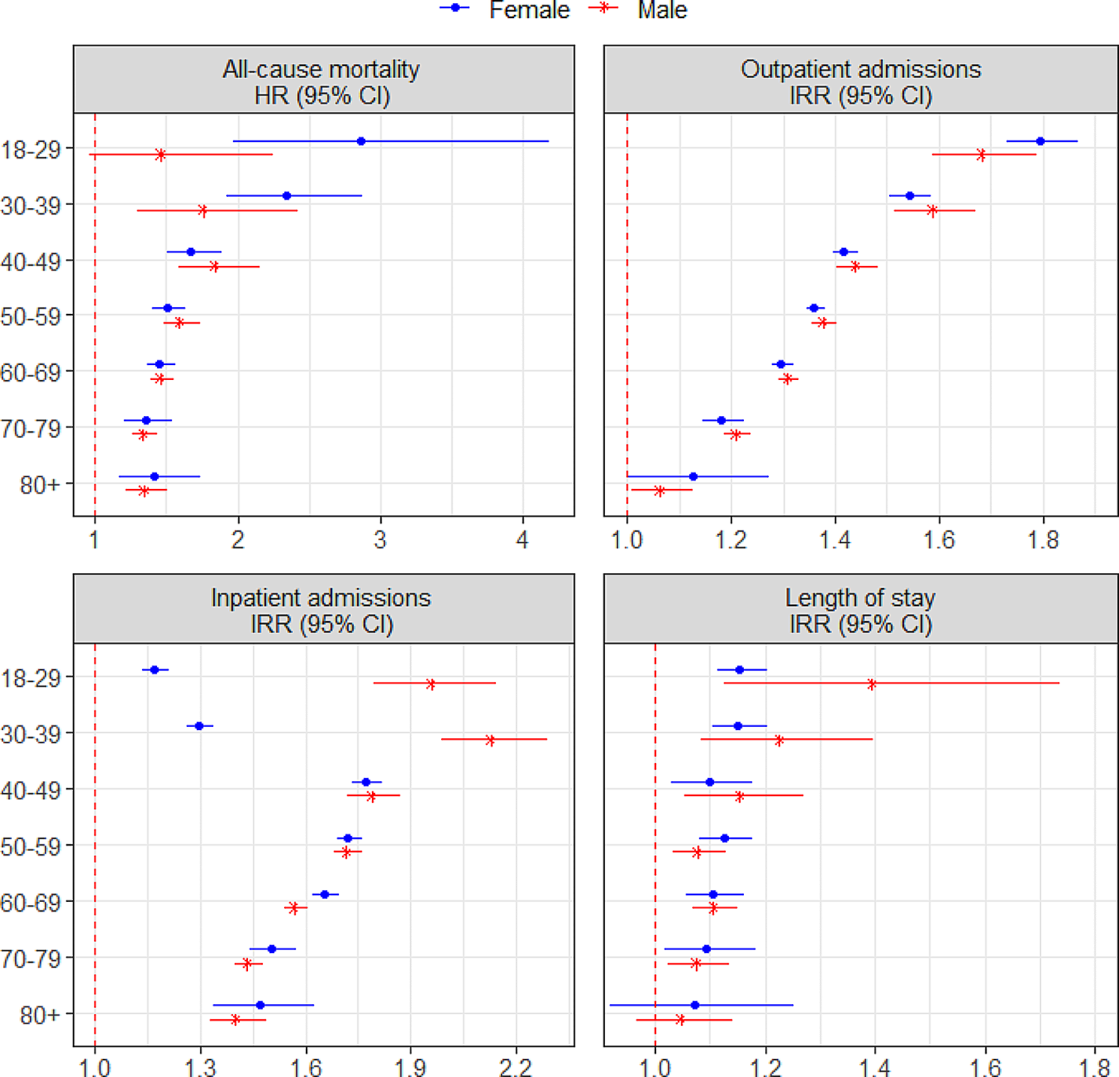
Forest plots depicting estimates from stratified, adjusted models for frailty in relation to all-cause mortality, inpatient and outpatient admissions and length of stay for inpatient admissions. The hazard ratio (HR) or incidence rate ratio (IRR) is relative to a 0.1-unit increase in frailty

Over the follow-up period, 6,951 deaths were observed. The cumulative incidence increased exponentially with frailty, where 5-year estimates for the low, mild, moderate and high groups were 0.5, 1.2, 3.5, and 7.9% (Fig. 2A). For the entire sample, a 0.1-unit increase in frailty was associated with a 1.87-fold increased hazard of death (95% CI = 1.83, 1.91) in univariable analysis, and a 1.47-fold increase (1.44, 1.51) when adjusted for age, sex, ethnicity, income, alcohol consumption and smoking status (5,934 events in 137,502 complete cases). However, hazard estimates were observed to differ quite substantially depending on the age group considered, and also for participant sex to a lesser extent (Fig. 3, upper left; Table 2; Supplemental Table 3). The hazard of death associated with frailty tended to decrease with age (supported by a significant age x frailty interaction (data not shown)) and was particularly high for women younger than 40; for example, the respective HR (95% CI) for 30–39 year old women and men was 2.34 (1.91, 2.86) and 1.76 (1.28, 2.41), as compared to 1.35 (1.20, 1.53) and 1.33 (1.25, 1.43) in 70–79 year old women and men. The hazard was dramatically higher in women aged 18–29 (2.86 [1.96, 4.18]) as compared to men (1.46 [0.95, 2.24]), although the error around these estimates was also higher, likely due to the relatively smaller observed number of deaths (50 vs. 42, respectively).

**Table 2:** A summary of estimates from stratified, adjusted models for frailty in relation to the outcomes all-cause mortality, inpatient and outpatient admissions over time, and length of stay for inpatient admissions. All estimates are relative to a 0.1-unit increase in frailty

		All-cause mortality	Outpatient admissions	Inpatient admissions	Length of stay
		HR (95% CI)	IRR (95% CI)	IRR (95% CI)	IRR (95% CI)
Women	18–29	2.86 (1.96, 4.18)	1.8 (1.73, 1.86)	1.17 (1.13, 1.21)	1.15 (1.11, 1.2)
	30–39	2.34 (1.91, 2.86)	1.54 (1.5, 1.58)	1.29 (1.26, 1.33)	1.15 (1.1, 1.2)
	40–49	1.67 (1.49, 1.87)	1.42 (1.39, 1.44)	1.77 (1.73, 1.81)	1.1 (1.03, 1.17)
	50–59	1.5 (1.39, 1.62)	1.36 (1.34, 1.38)	1.72 (1.69, 1.76)	1.13 (1.08, 1.17)
	60–69	1.45 (1.35, 1.56)	1.3 (1.28, 1.32)	1.65 (1.62, 1.69)	1.11 (1.05, 1.16)
	70–79	1.35 (1.2, 1.53)	1.18 (1.14, 1.22)	1.5 (1.44, 1.57)	1.09 (1.01, 1.18)
	80+	1.42 (1.16, 1.73)	1.12 (0.99, 1.27)	1.47 (1.33, 1.62)	1.07 (0.92, 1.25)
Men	18–29	1.46 (0.95, 2.24)	1.68 (1.59, 1.78)	1.96 (1.79, 2.14)	1.39 (1.12, 1.73)
	30–39	1.76 (1.28, 2.41)	1.59 (1.51, 1.67)	2.13 (1.98, 2.28)	1.23 (1.08, 1.39)
	40–49	1.84 (1.58, 2.14)	1.44 (1.4, 1.48)	1.79 (1.71, 1.86)	1.15 (1.05, 1.27)
	50–59	1.59 (1.47, 1.72)	1.38 (1.35, 1.4)	1.72 (1.68, 1.76)	1.08 (1.03, 1.13)
	60–69	1.46 (1.38, 1.54)	1.31 (1.29, 1.33)	1.57 (1.53, 1.6)	1.11 (1.07, 1.15)
	70–79	1.33 (1.25, 1.43)	1.21 (1.18, 1.24)	1.43 (1.39, 1.47)	1.08 (1.02, 1.13)
	80+	1.34 (1.2, 1.5)	1.06 (1.01, 1.12)	1.4 (1.32, 1.48)	1.05 (0.96, 1.14)

### Associations between frailty and healthcare utilization

A total of 270,005 hospital admissions were observed over the follow-up period, 177,186 of which were classified as outpatient (67%) and 92,819 as inpatient. For outpatient admissions, the mean cumulative incidence over 5 years in the low, mild, moderate and high frailty groups was 0.40, 0.67, 1.13, and 1.63 visits, respectively, whereas for inpatient admissions it was lower at 0.23, 0.31, 0.59, and 1.13 visits (Fig. 2B). In the entire sample, after adjusting for age, sex, ethnicity, marital status, education, income, rurality, region, alcohol consumption and smoking status (complete cases = 134,776, r^2^-outpatient = 0.377, r^2^-inpatient = 0.262), frailty was observed to be more strongly associated with the rate of inpatient admissions, where the incidence of inpatient admissions increased 1.60-times (95% CI = 1.59, 1.62) for every 0.1-unit increase in frailty, and outpatient visits only increased 1.35-times (1.34, 1.36). Although in both cases estimates generally decreased with age, as supported by a significant age x frailty interaction (data not shown), the difference in sex-specific patterns was notable (Fig. 3; Table 2; Supplemental Table 3). The rate of outpatient admissions associated with frailty decreased consistently with age, and generally, were slightly higher for men. The rate of inpatient admissions also decreased consistently with age after 40 years old, but were markedly different between sexes at younger ages (Fig. 2C). For men aged 18–39, the adjusted IRR was between 1.96 and 2.13, whereas for women it was only 1.17 and 1.29; this was actually lower than adults 80 years of age and older.

The mean of the number of days for a given inpatient admission across the entire sample was 3.4 ± 5.0. This increased with frailty, where the mean length of stay for the low, mild, moderate and high groups was 2.7, 3.3, 4.0, and 4.7 days, respectively. In a multivariable model, adjusting for the aforementioned covariates (complete cases = 78,020), the length of stay was observed to increase 1.12-times (95% CI = 1.10, 1.14) for every 0.1-unit increase in frailty. This association was mostly stable with age, and did not obviously differ between sexes (Fig. 3; Table 2). Notably larger estimates were observed for women aged 18–29 and even 30–39, although the error of these associations complicate their interpretation.

## Discussion

The primary goals of our study were two-fold. First, we sought to characterize how frailty was associated with health outcomes across a broad age spectrum. Given that frailty is commonly perceived as a geriatric syndrome that is unique to older adults, there is relatively little data pertaining to adults younger than 40 years of age. Second, we aimed to investigate how frailty was associated with healthcare utilization, which is relatively understudied in this area of the literature, although arguably one of the most important considerations for planning related to the delivery of healthcare services.

Our measure of frailty, based on the deficit accumulation model [14], increased with age, was higher in women of all ages, and was associated with socioeconomic and behavioral factors as previously shown [16]. Further, we show it to be significantly higher in Northern Ontario, which suffers from disparities in healthcare access [18]. The overall risk of all-cause mortality associated with frailty in our cohort was similar to that reported for Canadian adults [19], and tended to decrease with older age, which has also been reported in Chinese adults [9]. Although this association differed very little between sexes in adults over 40, which is supported by the literature [20], younger men, particularly those in the 18–29 age group, exhibited hazard estimates that were much lower than similarly aged women. While caution should be exercised given that relatively fewer deaths were observed at younger ages (i.e. for 18–29 year olds, 105 deaths in 26,070 participants), the overall trend does make sense when one considers the causes of death in youth. From 18 to 44 years old the leading causes of death in Canadian men are accidents and suicide, and in women it is predominantly cancer [21]. The impact of frailty on these events are likely to differ significantly: factors that are commonly considered in frailty indices, such as chronic conditions [22], obesity [23], inactivity [23] and mental illness [24], have all been associated with poor health outcomes in young women with cancer, whereas there is little evidence to suggest the same in young trauma patients [25]. Around the age of 45 the leading causes of death in both sexes are cancer- and heart-related [21] at which point our findings suggest no apparent sex-differences in the frailty-related risk in mortality.

To evaluate the association of frailty with healthcare utilization, we focused on the rate of hospital admissions and the length of inpatient stays. Overall, we found frailty to be significantly associated with both of these outcome measures, consistent with previous research [26]. In addition, frailty-associated rates were found to be higher for inpatient versus outpatient admissions, also consistent with previous research [27]. For outpatient admissions, there was a clear age-related reduction in the incidence rate associated with frailty and between the ages of 30 and 79 years, this was slightly lower in women as compared to men. While a similar age-related reduction was also apparent for inpatient admissions, there was little clear difference between sexes after age 40. However, at younger ages frailer women were much less likely to have an overnight stay as compared to frailer men. The incidence of inpatient admissions associated with frailty was lowest amongst women less than 40 as compared to any other strata. This may be attributed to relatively younger women being admitted for inpatient maternity services such as childbirth [28], which is unlikely to be related to frailty in any way.

Our study shows a clear trend in both all-cause mortality and hospital admissions where the magnitude of association with frailty decreases with participant age. There are a few plausible explanations for this trend. First, we may be observing a survivor effect in older birth cohorts, where only the most resilient or best supported frail older adults were able to participate in the Ontario Health Study. Second, overall healthcare service utilization, which includes consultation with a family physician [29], increases with age [30] and access to government supported preventative health programs tends to become available around the age of retirement. Regular access to a family physician alone reduces the need for acute or urgent hospital care [31] or the risk of catastrophic health outcomes [32] and would be expected to reduce the impact of frailty on health outcomes as well. Lastly, given that the effect of frailty on health outcomes likely plateaus with increasing magnitude [8, 33], one would expect hazard estimates in groups with relatively lower levels of frailty (i.e. younger adults) to be greater than groups where levels are inherently higher (i.e. older adults). Nonetheless, a critical takeaway of our findings is not that the association of frailty with mortality or healthcare utilization decreases with age, but that it is a significant factor in adults as young as 18–29.

Our study had significant strengths and some limitations. First, we employed a very large, population-based sample of adults over a broad age spectrum, for which we were able to characterize a combination of sociodemographics, lifestyle and health-related factors. Second, the health outcomes we investigated were obtained from administrative databases and could be linked for nearly all participants over a median follow-up of 7 years. Although our sample was large it likely omitted individuals who were relatively frailer than their peers such as those residing in long-term care facilities or from disadvantaged/marginalized populations that may not had access to the internet (a prerequisite for study enrollment). Also, the frailty index we developed was relatively small in terms of total number and diversity of items included and was based on self-reported data, which is more likely to result in misclassification and suffer from missingness due to recollection bias and/or the sensitive nature of some questions [34]. The index did, however, exhibit face validity in its associations with participant sociodemographic and lifestyle factors.

In conclusion, our study showed that frailty in terms of deficit accumulation is an important health factor regardless of age or sex. Given that the prevalence and severity of frailty is increasing even at younger ages [35], our findings suggest that routine frailty screening at the level of primary care could pay significant dividends with regards to reducing future healthcare burden. As previously argued by others [36], this would be particularly true for younger individuals experiencing socioeconomic deprivation given that their frailty levels tend to increase more steeply over time [37]. Hence, monitoring health deficit accumulation by family health teams for patients even in their 20 or 30 s would aid in the efficient delivery of preventative health interventions such as mental health screening, smoking cessation and vaccination. For example, consistent communication with a family physician significantly improves vaccine uptake amongst younger or older adults [38, 39], and reduces hospital visits in people with complex health needs [40]. From a conservative perspective, purposeful assessment of frailty and targeted social and health interventions to reduce the impact of frailty before the age of 60 years is supported through the findings of this study.

### Electronic supplementary material

Below is the link to the electronic supplementary material.

Supplementary Material 1

## Data Availability

Data from the Ontario Health Study and ICES is available to researchers who meet the eligibility requirements.
