# Supplementary material for: Age- and sex-specific associations of frailty with mortality and healthcare utilization in community-dwelling adults from Ontario, Canada
Source: BMC Geriatr. 2024 Mar 4;24:223. doi: 10.1186/s12877-024-04842-4 (PMC10913570; doi:10.1186/s12877-024-04842-4)
Supplement: Supplementary file 1 — Supplementary Material 1 [file 12877_2024_4842_MOESM1_ESM.docx]

# Supplemental tables

**Supplemental Table 1:** A summary of the 30 items included in the frailty index, including scoring convention, classification and frequency within the study cohort.

|  |  |  | **OHS (n=161,149)** |
| --- | --- | --- | --- |
| **Self-rated health and other (n=6)** | | | |
|  | General health | | |
|  | 0 | Very good or excellent | 90828 (56.4%) |
|  | 0.5 | Good | 51616 (32.0%) |
|  | 1 | Poor or fair | 18539 (11.5%) |
|  | Missing | | 166 (0.1%) |
|  | Vision | | |
|  | 0 | Very good or excellent | 87146 (54.1%) |
|  | 0.5 | Good | 30696 (19.0%) |
|  | 1 | Poor or fair | 9758 (6.1%) |
|  | Missing | | 33549 (20.8%) |
|  | Body-mass index (BMI)^#^ | | |
|  | 0 | Normal | 54628 (33.9%) |
|  | 0.5 | Overweight | 49165 (30.5%) |
|  | 1 | Underweight or Obese | 34972 (21.7%) |
|  | Missing | | 22384 (13.9%) |
|  | Sleep (hrs/week) | | |
|  | 0 | ≥ 7 hours | 114089 (70.8%) |
|  | 0.5 | 5-7 hours | 39385 (24.4%) |
|  | 1 | < 5 hours | 3192 (2.0%) |
|  | Missing | | 4483 (2.8%) |
|  | Poor sleep (frequency) | | |
|  | 0 | Never or part of the time | 77328 (48.0%) |
|  | 0.5 | Some of the time | 53046 (32.9%) |
|  | 1 | Most or all of the time | 30063 (18.7%) |
|  | Missing | | 712 (0.4%) |
|  | Lives alone (not married and no children living at home) | | |
|  | 0 | No | 130771 (81.1%) |
|  | 1 | Yes | 27069 (16.8%) |
|  | Missing | | 3309 (2.1%) |
| **Physical activity (n=3)** | | | |
|  | Walking (/week) | | |
|  | 0 | 6-7 days | 67096 (41.6%) |
|  | 0.5 | 3-5 days | 51789 (32.1%) |
|  | 1 | 0-2 days | 39892 (24.8%) |
|  | Missing | | 2372 (1.5%) |
|  | Moderate activity (/week) | | |
|  | 0 | Some time | 80962 (50.2%) |
|  | 1 | Not at all | 61630 (38.2%) |
|  | Missing | | 18557 (11.5%) |
|  | Vigorous activity (/week) | | |
|  | 0 | Some time | 74146 (46.0%) |
|  | 1 | Not at all | 77946 (48.4%) |
|  | Missing | | 9057 (5.6%) |
| **Disability (n=2)** | | | |
|  | Unable to work due to illness | | |
|  | 0 | No | 152430 (94.6%) |
|  | 1 | Yes | 5747 (3.6%) |
|  | Missing | | 2972 (1.8%) |
|  | Unable to stand without assistance | | |
|  | 0 | No | 156516 (97.1%) |
|  | 1 | Yes | 1081 (0.7%) |
|  | Missing | | 3552 (2.2%) |
| **Chronic conditions (n=16)** | | | |
|  | Arthritis ever | | |
|  | 0 | No | 125572 (77.9%) |
|  | 1 | Yes | 32902 (20.4%) |
|  | Missing | | 2675 (1.7%) |
|  | Asthma ever | | |
|  | 0 | No | 136330 (84.6%) |
|  | 1 | Yes | 22643 (14.1%) |
|  | Missing | | 2176 (1.4%) |
|  | Cancer ever | | |
|  | 0 | No | 145141 (90.1%) |
|  | 1 | Yes | 14771 (9.2%) |
|  | Missing | | 1237 (0.8%) |
|  | Heart disease ever | | |
|  | 0 | No | 138240 (85.8%) |
|  | 1 | Yes | 4956 (3.1%) |
|  | Missing | | 17953 (11.1%) |
|  | Chronic obstructive pulmonary disease ever | | |
|  | 0 | No | 156345 (97.0%) |
|  | 1 | Yes | 2214 (1.4%) |
|  | Missing | | 2590 (1.6%) |
|  | Crohn's disease ever | | |
|  | 0 | No | 159543 (99.0%) |
|  | 1 | Yes | 1040 (0.6%) |
|  | Missing | | 566 (0.4%) |
|  | Depression ever | | |
|  | 0 | No | 140309 (87.1%) |
|  | 1 | Yes | 17505 (10.9%) |
|  | Missing | | 3335 (2.1%) |
|  | Diabetes ever | | |
|  | 0 | No | 148756 (92.3%) |
|  | 1 | Yes | 10467 (6.5%) |
|  | Missing | | 1926 (1.2%) |
|  | High blood pressure ever | | |
|  | 0 | No | 123553 (76.7%) |
|  | 1 | Yes | 34649 (21.5%) |
|  | Missing | | 2947 (1.8%) |
|  | Irritable bowel syndrome ever | | |
|  | 0 | No | 146798 (91.1%) |
|  | 1 | Yes | 12212 (7.6%) |
|  | Missing | | 2139 (1.3%) |
|  | Heart attack ever | | |
|  | 0 | No | 156395 (97.1%) |
|  | 1 | Yes | 3278 (2.0%) |
|  | Missing | | 1476 (0.9%) |
|  | Multiple sclerosis ever | | |
|  | 0 | No | 159779 (99.1%) |
|  | 1 | Yes | 827 (0.5%) |
|  | Missing | | 543 (0.3%) |
|  | Osteoporosis ever | | |
|  | 0 | No | 152807 (94.8%) |
|  | 1 | Yes | 6816 (4.2%) |
|  | Missing | | 1526 (0.9%) |
|  | Parkinson's disease ever | | |
|  | 0 | No | 152395 (94.6%) |
|  | 1 | Yes | 7622 (4.7%) |
|  | Missing | | 1132 (0.7%) |
|  | Stroke ever | | |
|  | 0 | No | 157719 (97.9%) |
|  | 1 | Yes | 1726 (1.1%) |
|  | Missing | | 1704 (1.1%) |
|  | Ulcerative colitis ever | | |
|  | 0 | No | 158819 (98.6%) |
|  | 1 | Yes | 1569 (1.0%) |
|  | Missing | | 761 (0.5%) |
| **Chronic conditions, other (n=3)** | | | |
|  | Radiation or chemotherapy ever | | |
|  | 0 | No | 154850 (96.1%) |
|  | 1 | Yes | 5062 (3.1%) |
|  | Missing | | 1237 (0.8%) |
|  | Family history of dementia | | |
|  | 0 | No | 113702 (70.6%) |
|  | 1 | Yes | 15277 (9.5%) |
|  | Missing | | 32170 (20.0%) |
|  | Medications* | | |
|  | 0 | None | 145283 (90.2%) |
|  | 0.2 | 1 | 8773 (5.4%) |
|  | 0.4 | 2 | 2623 (1.6%) |
|  | 0.6 | 3 | 1521 (0.9%) |
|  | 0.8 | 4 | 1044 (0.6%) |
|  | 1 | 5 or more | 1905 (1.2%) |
|  | Missing | | 0 (0%) |

^#^ BMI was calculated from self-reported height and weight

* questionnaires did not allow participants to specify no medication use.

**Supplemental Table 2:** Associations of frailty with sociodemographic and lifestyle characteristics in the sample population.

|  |  | **Crude beta [95% CI]** | **Adjusted beta [95% CI]** |
| --- | --- | --- | --- |
| Age | 18-29 | - | - |
|  | 30-39 | 0.019 (0.018, 0.021) | 0.031 (0.029, 0.032) |
|  | 40-49 | 0.038 (0.037, 0.040) | 0.049 (0.048, 0.051) |
|  | 50-59 | 0.062 (0.061, 0.063) | 0.071 (0.070, 0.073) |
|  | 60-69 | 0.079 (0.078, 0.081) | 0.087 (0.085, 0.088) |
|  | 70-79 | 0.098 (0.096, 0.101) | 0.103 (0.101, 0.105) |
|  | 80+ | 0.121 (0.117, 0.125) | 0.125 (0.121, 0.130) |
| Sex | Female | - | - |
|  | Male | -0.006 (-0.007, -0.005) | -0.007 (-0.008, -0.006) |
| Ethnicity | Not white | - | - |
|  | White | 0.015 (0.013, 0.016) | 0.005 (0.004, 0.006) |
| Marital | Never married | - | - |
|  | Previously married | 0.061 (0.059, 0.062) | 0.006 (0.005, 0.008) |
|  | Married | 0.007 (0.006, 0.008) | -0.020 (-0.021, -0.019) |
| Education | Less than diploma | - | - |
|  | Diploma | -0.011 (-0.012, -0.010) | -0.004 (-0.005, -0.003) |
|  | Bachelor | -0.036 (-0.037, -0.035) | -0.013 (-0.014, -0.012) |
|  | Graduate | -0.037 (-0.038, -0.036) | -0.017 (-0.018, -0.016) |
| Income | Less than 50K | - | - |
|  | 50-100K | -0.020 (-0.022, -0.019) | -0.016 (-0.017, -0.015) |
|  | 100-150K | -0.038 (-0.039, -0.036) | -0.023 (-0.025, -0.022) |
|  | 150K+ | -0.050 (-0.052, -0.049) | -0.030 (-0.031, -0.028) |
| Geography | Urban | - | - |
|  | Rural | 0.005 (0.004, 0.006) | -0.003 (-0.005, -0.002) |
| Region | South | - | - |
|  | North | 0.012 (0.010, 0.014) | 0.005 (0.004, 0.007) |
| Alcohol consumption | <1/Month | - | - |
|  | 1-3/Month | -0.023 (-0.024, -0.022) | -0.016 (-0.017, -0.014) |
|  | 1-3/Week | -0.032 (-0.033, -0.031) | -0.027 (-0.028, -0.026) |
|  | 4-7/Week | -0.015 (-0.016, -0.014) | -0.029 (-0.031, -0.028) |
| Smoking status [Pack years] | Never | - | - |
|  | Former [<10] | 0.018 (0.017, 0.019) | 0.009 (0.008, 0.010) |
|  | Current [<10] | 0.011 (0.010, 0.013) | 0.016 (0.014, 0.017) |
|  | Former [10+] | 0.063 (0.062, 0.064) | 0.033 (0.031, 0.034) |
|  | Current [10+] | 0.061 (0.060, 0.063) | 0.034 (0.032, 0.036) |
| Observations | 134776 |  |  |
| R^2^ / R^2^ adjusted | 0.245 / 0.245 | | |

Note: the first category (i.e. “-“) represents the reference.

**Supplemental Table 3:** Crude and adjusted associations between frailty (per 0.1-unit increase) and study outcomes.

|  |  |  | **Crude** | | | **Adjusted** | | |
| --- | --- | --- | --- | --- | --- | --- | --- | --- |
|  |  |  | **n** | **e** | **Est. (95% CI)** | **n** | **e** | **Est. (95% CI)** |
| **All-cause mortality HR (95% CI)** | Women | 18-29 | 17631 | 58 | 3.22 (2.38, 4.36) | 14209 | 50 | 2.86 (1.96, 4.18) |
|  |  | 30-39 | 18133 | 148 | 2.34 (1.99, 2.77) | 15644 | 125 | 2.34 (1.91, 2.86) |
|  |  | 40-49 | 20951 | 318 | 1.81 (1.65, 1.98) | 17839 | 275 | 1.67 (1.49, 1.87) |
|  |  | 50-59 | 23375 | 785 | 1.64 (1.54, 1.74) | 19933 | 656 | 1.5 (1.39, 1.62) |
|  |  | 60-69 | 13525 | 922 | 1.59 (1.5, 1.69) | 11478 | 767 | 1.45 (1.35, 1.56) |
|  |  | 70-79 | 2833 | 427 | 1.41 (1.28, 1.56) | 2372 | 348 | 1.35 (1.2, 1.53) |
|  |  | 80+ | 409 | 172 | 1.41 (1.18, 1.68) | 342 | 145 | 1.42 (1.16, 1.73) |
|  | Men | 18-29 | 8439 | 47 | 1.68 (1.2, 2.36) | 6803 | 42 | 1.46 (0.95, 2.24) |
|  |  | 30-39 | 9340 | 80 | 2.01 (1.58, 2.57) | 8072 | 66 | 1.76 (1.28, 2.41) |
|  |  | 40-49 | 12226 | 276 | 1.92 (1.71, 2.16) | 10627 | 237 | 1.84 (1.58, 2.14) |
|  |  | 50-59 | 15124 | 744 | 1.72 (1.62, 1.83) | 13231 | 640 | 1.59 (1.47, 1.72) |
|  |  | 60-69 | 13625 | 1356 | 1.59 (1.52, 1.67) | 12082 | 1189 | 1.46 (1.38, 1.54) |
|  |  | 70-79 | 4629 | 1123 | 1.39 (1.31, 1.48) | 4091 | 980 | 1.33 (1.25, 1.43) |
|  |  | 80+ | 909 | 495 | 1.35 (1.23, 1.49) | 779 | 414 | 1.34 (1.2, 1.5) |
| **Outpatient admissions IRR (95% CI)** | Women | 18-29 | 17631 | 7352 | 1.86 (1.81, 1.92) | 13854 | 5890 | 1.8 (1.73, 1.86) |
|  |  | 30-39 | 18133 | 12091 | 1.58 (1.55, 1.61) | 15331 | 10163 | 1.54 (1.5, 1.58) |
|  |  | 40-49 | 20951 | 20666 | 1.42 (1.4, 1.44) | 17510 | 17172 | 1.42 (1.39, 1.44) |
|  |  | 50-59 | 23375 | 30811 | 1.34 (1.32, 1.35) | 19514 | 25529 | 1.36 (1.34, 1.38) |
|  |  | 60-69 | 13525 | 23198 | 1.29 (1.28, 1.31) | 11267 | 19312 | 1.3 (1.28, 1.32) |
|  |  | 70-79 | 2833 | 5638 | 1.17 (1.14, 1.21) | 2330 | 4704 | 1.18 (1.14, 1.22) |
|  |  | 80+ | 409 | 583 | 1.16 (1.05, 1.28) | 335 | 425 | 1.12 (0.99, 1.27) |
|  | Men | 18-29 | 8439 | 2287 | 1.65 (1.57, 1.72) | 6586 | 1814 | 1.68 (1.59, 1.78) |
|  |  | 30-39 | 9340 | 4088 | 1.54 (1.47, 1.6) | 7906 | 3491 | 1.59 (1.51, 1.67) |
|  |  | 40-49 | 12226 | 9432 | 1.5 (1.46, 1.53) | 10404 | 8087 | 1.44 (1.4, 1.48) |
|  |  | 50-59 | 15124 | 19525 | 1.38 (1.36, 1.4) | 13011 | 16729 | 1.38 (1.35, 1.4) |
|  |  | 60-69 | 13625 | 27319 | 1.3 (1.28, 1.32) | 11917 | 24024 | 1.31 (1.29, 1.33) |
|  |  | 70-79 | 4629 | 12045 | 1.2 (1.18, 1.23) | 4038 | 10569 | 1.21 (1.18, 1.24) |
|  |  | 80+ | 909 | 2151 | 1.08 (1.03, 1.13) | 773 | 1853 | 1.06 (1.01, 1.12) |
| **Inpatient admissions IRR (95% CI)** | Women | 18-29 | 17631 | 13075 | 1.18 (1.14, 1.21) | 13854 | 10728 | 1.17 (1.13, 1.21) |
|  |  | 30-39 | 18133 | 11443 | 1.24 (1.21, 1.27) | 15331 | 9608 | 1.29 (1.26, 1.33) |
|  |  | 40-49 | 20951 | 7525 | 1.86 (1.82, 1.89) | 17510 | 6216 | 1.77 (1.73, 1.81) |
|  |  | 50-59 | 23375 | 11228 | 1.81 (1.78, 1.83) | 19514 | 9391 | 1.72 (1.69, 1.76) |
|  |  | 60-69 | 13525 | 9984 | 1.73 (1.7, 1.76) | 11267 | 8275 | 1.65 (1.62, 1.69) |
|  |  | 70-79 | 2833 | 3205 | 1.64 (1.59, 1.7) | 2330 | 2529 | 1.5 (1.44, 1.57) |
|  |  | 80+ | 409 | 761 | 1.4 (1.29, 1.52) | 335 | 602 | 1.47 (1.33, 1.62) |
|  | Men | 18-29 | 8439 | 968 | 1.81 (1.7, 1.91) | 6586 | 752 | 1.96 (1.79, 2.14) |
|  |  | 30-39 | 9340 | 1603 | 2.31 (2.18, 2.44) | 7906 | 1298 | 2.13 (1.98, 2.28) |
|  |  | 40-49 | 12226 | 3607 | 2.02 (1.95, 2.08) | 10404 | 3008 | 1.79 (1.71, 1.86) |
|  |  | 50-59 | 15124 | 8431 | 1.8 (1.77, 1.83) | 13011 | 7272 | 1.72 (1.68, 1.76) |
|  |  | 60-69 | 13625 | 12444 | 1.63 (1.6, 1.66) | 11917 | 10961 | 1.57 (1.53, 1.6) |
|  |  | 70-79 | 4629 | 6703 | 1.48 (1.45, 1.52) | 4038 | 5826 | 1.43 (1.39, 1.47) |
|  |  | 80+ | 909 | 1842 | 1.44 (1.37, 1.51) | 773 | 1555 | 1.4 (1.32, 1.48) |
| **Length of stay IRR (95% CI)** | Women | 18-29 | 13075 |  | 1.15 (1.11, 1.2) | 10728 |  | 1.15 (1.11, 1.2) |
|  |  | 30-39 | 11443 |  | 1.2 (1.16, 1.24) | 9608 |  | 1.15 (1.1, 1.2) |
|  |  | 40-49 | 7525 |  | 1.14 (1.09, 1.2) | 6216 |  | 1.1 (1.03, 1.17) |
|  |  | 50-59 | 11228 |  | 1.14 (1.1, 1.18) | 9391 |  | 1.13 (1.08, 1.17) |
|  |  | 60-69 | 9984 |  | 1.17 (1.11, 1.23) | 8275 |  | 1.11 (1.05, 1.16) |
|  |  | 70-79 | 3205 |  | 1.14 (1.07, 1.21) | 2529 |  | 1.09 (1.01, 1.18) |
|  |  | 80+ | 761 |  | 1.05 (0.92, 1.21) | 602 |  | 1.07 (0.92, 1.25) |
|  | Men | 18-29 | 968 |  | 1.06 (0.54, 2.07) | 752 |  | 1.39 (1.12, 1.73) |
|  |  | 30-39 | 1603 |  | 1.22 (1.09, 1.36) | 1298 |  | 1.23 (1.08, 1.39) |
|  |  | 40-49 | 3607 |  | 1.23 (1.13, 1.34) | 3008 |  | 1.15 (1.05, 1.27) |
|  |  | 50-59 | 8431 |  | 1.12 (1.07, 1.17) | 7272 |  | 1.08 (1.03, 1.13) |
|  |  | 60-69 | 12444 |  | 1.15 (1.11, 1.19) | 10961 |  | 1.11 (1.07, 1.15) |
|  |  | 70-79 | 6703 |  | 1.07 (1.02, 1.12) | 5826 |  | 1.08 (1.02, 1.13) |
|  |  | 80+ | 1842 |  | 1.02 (0.95, 1.1) | 1555 |  | 1.05 (0.96, 1.14) |

Est. refers to each model estimate, listed under each given outcome.

n and e, the number of participants and outcome events observed, respectively, within each sex- and age-strata. Note, the number of participants (n) and event count (e) represent the same value, which is the number of inpatient admissions within a given strata; hence, no values are shown for e.
